# Low molecular weight chitosan oligosaccharides form stable complexes with human lactoferrin

**DOI:** 10.1002/2211-5463.13722

**Published:** 2023-10-30

**Authors:** Juan Li, Shimin Guan, Baoguo Cai, Qianqian Li, Shaofeng Rong

**Affiliations:** ^1^ Department of Bioengineering, School of Perfume and Aroma Technology Shanghai Institute of Technology Shanghai China

**Keywords:** chitosan oligosaccharide, fluorescence quenching, human lactoferrin, interaction, molecular docking, UV–Vis spectra

## Abstract

Proteins in tears, including human lactoferrin (HLF), can be deposited and denatured on contact lenses, increasing the risk of microbial cell attachment to the lens and ocular complications. The surfactants currently used in commercial contact lens care solutions have low clearance ability for tear proteins. Chitosan oligosaccharide (COS) binds to a variety of proteins and has potential for use in protein removal, especially in contact lens care solutions. Here, we analyzed the interaction mechanism of COSs hydrolyzed from chitosan from different resources with HLF. The molecular weights (MWs) and concentrations of COSs were key factors for the formation of COS–HLF complexes. Lower MWs of COSs could form more stable COS–HLF complexes. COS from *Aspergillus ochraceus* had a superior effect on HLF compared with COS from shrimp and crab shell with the same MWs. In conclusion, COSs could bind to and cause a conformational change in HLF. Therefore, COSs, especially those with low MWs, have potential as deproteinizing agents in contact lens care solution.

## Graphical abstract

Proteins in tears, including human lactoferrin (HLF), can be deposited and denatured on contact lenses. Here, we demonstrated chitosan oligosaccharide (COS) can bind to and cause a conformational change in HLF. The molecular weights and concentrations of COSs were key factors for the formation of COS–HLF complexes. The crystalline structures of COSs had less impact on COS–HLF complex formation.

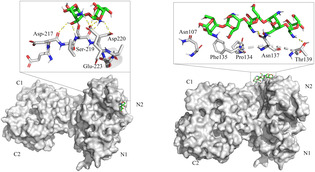

**Abbreviations**
COS: Chitosan oligosaccharide
GlcN: d‐glucosamine
GlcNAc: N‐acetyl d‐glucosamine
HLF: human lactoferrin
MWs: molecular weights

Human tear is complex mixture of proteins, lipids, metabolites, electrolytes, and some small organic molecules [1]. Over 400 proteins have been identified in human tears [2]. The total range of protein concentrations can be influenced by contact lens wear [3, 4] and age [5]. Proteins in tears can be deposited and denatured on contact lenses [6]. This situation increases the risk of microbial cell attachment to the lens and causes various ocular complications [7, 8, 9]. For example, it causes macropapillary conjunctivitis, which is the most common ocular complication in contact lens wearers [10, 11, 12].

Lactoferrin is one of the most abundant protein in human tear. The average concentration of lactoferrin is around 2 g·L^−1^, accounting for around 25% of the total tear proteins [13, 14]. Lactoferrin is a monomeric protein consisting of 691 amino acids [15]. The structure of lactoferrin consists of two globular lobules, a C‐lobe and an N‐lobe. Each globular lobule is made up of two domains named C1, C2 and N1, N2. Studies showed that lactoferrin attached to contact lens is more difficult to remove compared with lysozyme [16]. Therefore, removing lactoferrin deposits from contact lenses is essential to avoid ocular complications.

The surfactants currently used in commercial contact lens care solutions are mainly poloxamer (F127), polyethylene glycol‐400 (PEG‐400), and polyoxyethylene castor oil (EL‐35). However, these surfactants have low clearance ability for tear proteins [17, 18]. In addition, the PEG‐400 has potential side effects such as vision loss, cataract formation, retinal degeneration, and detachment after PEG‐400 injection in the retina or vitreous [19, 20].

Chitosan oligosaccharide (COS) is composed of d‐glucosamine (GlcN) and N‐acetyl d‐glucosamine (GlcNAc) linked by β‐1,4‐glycosidic bonds between them [21]. The average molecular weights (MWs) of COS are less than 3.9 kDa with a degree of polymerization between 2 and 20 [22, 23]. COS has good water solubility [24, 25], biocompatibility [26], low viscosity [27], low allergenicity, and cytotoxicity [28]. COS and chitosan (high degree of polymerization) can bind to a variety of proteins (e.g., serum albumin and lactoferrin) to form complexes that alter the conformation of the protein and affect its function [29, 30, 31, 32, 33]. Therefore, COS has potential applications for protein removal, especially in contact lens care solutions. However, limited research has been conducted on the interaction mechanism between lactoferrin and COS with different MWs and sources.

This paper aims to study the mechanism of interaction between COS and lactoferrin, and to provide theoretical support for the addition of COS as a descaling agent into contact lens care solutions.

## Materials and methods

### Materials

Recombinant human lactoferrin (HLF) was purchased from Wuhan Heyuan Biotechnology Co., Ltd (Wuhan, Hubei Province, China). Three COSs, named COS1, COS2, and COS3 (Table 1), were prepared by enzymatic hydrolysis using a recombinant chitosanase expressed in our laboratory [34]. Among them, COS1 was a mixture of chitobiose (the degree of polymerization was 2) and chitotriose (the degree of polymerization was 3), where the proportion of chitobiose was higher.

**Table 1:** Main parameters of the three COSs used in the experiment.

Name	Source	Main substances^a^	Average MW
COS1	Shrimp and crab shells	Chitobiose Chitotriose (mainly)	346.369 531.823
COS2	Shrimp and crab shells	Chitopentaose	816.107
COS3	Cell wall of *Aspergillus ochraceus*	Chitopentaose	813.336

^a^ Chitobiose, chitotriose, and chitopentaose are COSs with degree of polymerization 2, 3, and 5, respectively.

### Preparation of solutions

The HLF was dissolved in 0.9% NaCl with a final concentration of 0.02 g·L^−1^. Each COS was first dissolved in 0.9% NaCl with a concentration and prepared for a series dilution. The HLF and COS solutions were mixed to obtain a series of final concentration ratios (COS : HLF) of 1 : 8, 1 : 4, 1 : 2, 1 : 1, 2 : 1, and 4 : 1, and then, the final concentrations of COS reached 0.0025, 0.005, 0.01, 0.02, 0.04, and 0.08 g·L^−1^, respectively. All the mixtures were incubated for 30 min. Furthermore, all the experiments mentioned below were performed at 25 °C unless stated otherwise.

### Binding parameters between COS and HLF

The binding parameters between COS and HLF were further investigated using Stern–Volmer equation [35]:

(1) $$\frac{F_{0}}{F}=1+K_{\mathrm{sv}}\left[Q\right]=1+K_{q}\tau_{0}\left[Q\right],$$

where $F_{0}$ and F are the fluorescence intensities before and after the addition of the quencher, respectively; $\left[Q\right]$ is the quencher concentration; $K_{\mathrm{sv}}$ is the Stern–Volmer quenching constant; $K_{q}$ is the bimolecular quenching constant; and $\tau_{0}$ is the unquenched lifetime, which is 10^8^ s.

The binding constants and number of binding sites for COS–HLF complex could be generally obtained by modified Stern–Volmer equation [36]:

(2) $$\mathrm{lg}\left[\frac{F_{0}-F}{F}\right]=\mathrm{lg}K_{a}+n\mathrm{lg}\left[Q\right],$$

where $K_{a}$ and *n* are the binding constant and the number of binding sites, respectively. Then, the number of binding sites in COS‐HLF complex could be determined from Eq. (2).

Thermodynamic parameters are important to determine the binding mode of the COS–HLF complex. When the temperature ranges do not vary much, enthalpy change can be considered a constant [30]. The Van't Hoff formula is usually used for the calculation [36].

(3) $$\ln\left(\frac{K_{2}}{K_{1}}\right)=-\frac{\Delta H}{R}\left(\frac{1}{T_{2}}-\frac{1}{T_{1}}\right),$$

(4) ΔG=ΔH−TΔS,

(5) $$\Delta G=-\mathrm{RT}\ln K_{a},$$

where ΔH, ΔG, and ΔS represent the enthalpy change, the free energy change, and the entropy change, respectively. R is the gas constant (8.314 J·mol^−1^·K^−1^), T is the Kelvin temperature, and $K_{a}$ represents the binding constant at the corresponding temperature. The type of interaction force between COS and HLF can be determined using Eqs. (3), (4), and (5).

### UV–Vis absorption experiment

The absorbances of HLF in the COS–HLF mixtures mentioned in Preparation of solutions section were measured on a UV‐2600 UV–Vis spectrometer (Shimadzu, Japan) in a wavelength ranging from 190 nm to 500 nm and a resolution of 0.5 nm.

### Fluorescence detection

The fluorescence spectra of HLF in the COS‐HLF mixtures were measured using an RF‐5301PC fluorophotometer (Shimadzu, Japan) at 25 °C and 37 °C, respectively. The excitation wavelength was 280 nm, and the scanning wavelength was 250–500 nm. The fluorescence excitation and emission slit widths were 5.0 nm with a sampling interval of 1.0 nm.

### Molecular docking

Molecular docking study was performed to determine the binding sites on HLF and the binding energy of protein–ligand complex. The 3D molecular model of HLF with PDB ID 1FCK was obtained from Protein Data Bank (http://www.rcsb.org) with a resolution of 2.20 Å. The structure of COSs (chitobiose and chitopentaose) as ligands were generated in ChemDraw 3D. For construction of HLF, the pymol software was first used to remove the free water and the bound ions. Then, hydrogen atoms were added to the protein structure. The docking studies were performed with the autodock 4.0 software [36]. Briefly, a grid box was created to contain the entire HLF molecule. HLF was held rigid, and all the torsional bonds of COS were taken as free during docking calculations. Lamarckian genetic algorithm was chosen as the docking algorithm. The GA population size was 50. The number of evaluations and generations were set to 3 000 000 and 30 000, respectively. The docking program was run to obtain the binding conformation of ligand and receptor. Finally, the interaction between HLF and COS was analyzed using pymol software.

### Data analysis

All the experiments were conducted three times. Data were shown as the mean ± standard of three parallel experiments (*N* = 3). Statistical analysis was carried out by one‐way ANOVA using origin 2018. Differences in means were considered significant when the *P* < 0.05.

## Results

### UV–Vis spectra showing structural changes in HLF

**Fig. 1:**
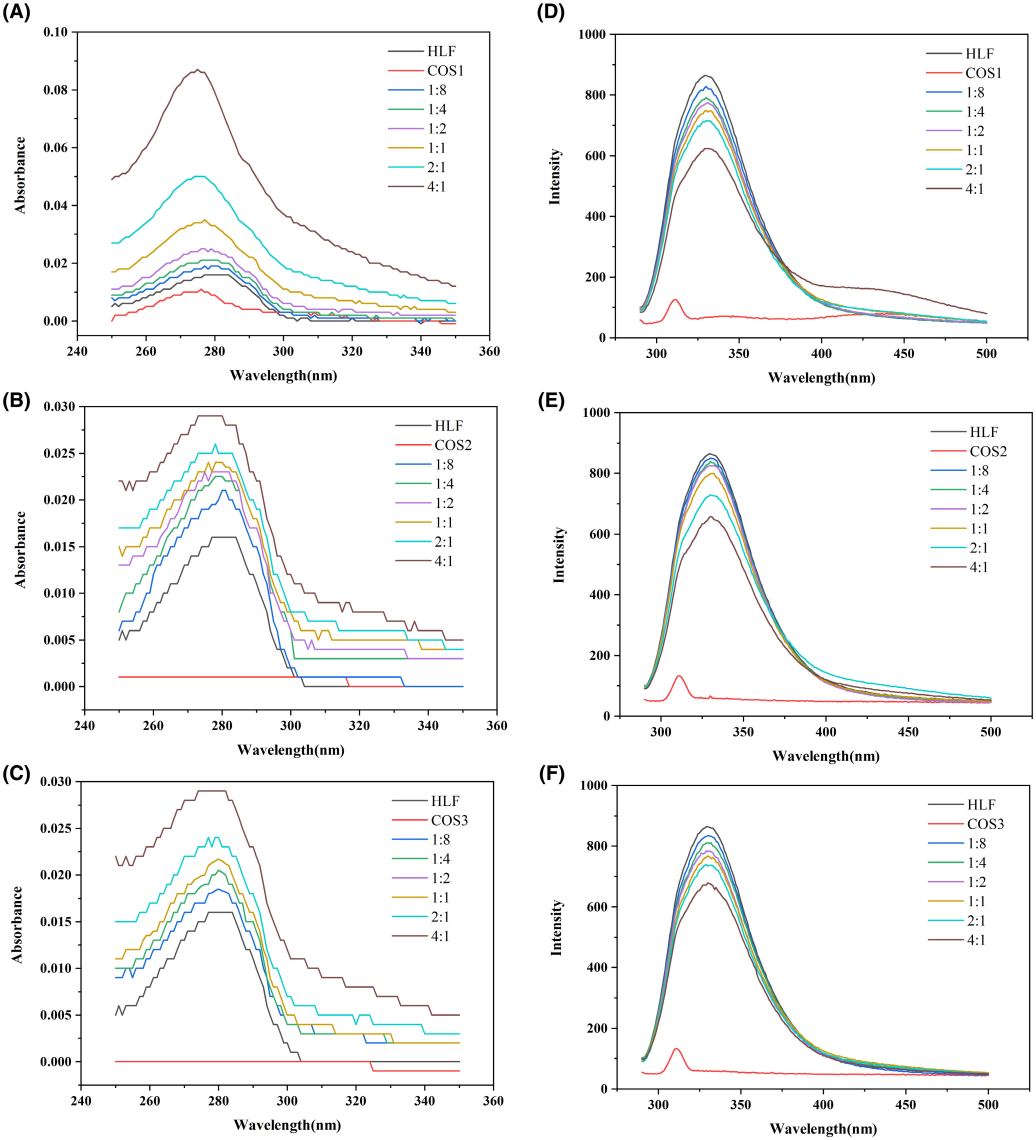
UV–Vis spectra (A–C) and fluorescence spectra (D–F) of complexes COS1–HLF, COS2–HLF, and COS3–HLF in solution with different HLF and COS mixing ratios. (A) and (D), COS1–HLF; (B) and (E), COS2–HLF; (C) and (F), COS3–HLF. Experiments were carried out in 0.9% NaCl solution at 298.15 K (Kelvins, for 25 °C). The HLF concentration was 0.02 g·L^−1^. The ratio of COS to HLF concentrations (COS:HLF) was listed in the diagrams.

The structural effects of three COSs (COS1, COS2, and COS3) on HLF at different concentrations were investigated using HLF as a model protein. Figure 1A–C show the three COSs had minute UV absorptions. The UV–Vis absorption spectra of the three COS‐HLF complexes (COS1‐HLF, COS2‐HLF, and COS3‐HLF) were affected by the COS concentrations. The higher the concentration of COS, the higher the UV absorption intensities of the COS–HLF complexes. Therefore, the UV absorption intensities were positively correlated with the concentrations of COS. For COS1, the maximum UV absorption of HLF at 280 nm was 0.016, whereas the absorption of the complex reached 0.087 when the final concentration ratios of COS1:HLF was 4 : 1, as shown in Fig. 1A. The UV absorptions of COS2–HLF, as shown in Fig. 1B, and COS3–HLF, as shown in Fig. 1C, were around 0.04.

When the concentration ratios of COS1 : HLF were 2 : 1 and 4 : 1, the wavelength of UV absorption peaks was blue shifted from 280 nm to 275 nm. The results were probably due to the reduced hydrophobicity of HLF under these concentrations. However, blue or red shifts in the COS2‐HLF and COS3‐HLF complexes were not discovered. According to these data, the effect of COS1 for the structure changes in HLF was higher than those of COS2 and COS3. Chitobiose and chitotriose had more influence on the structure changes for HLF. The source of COS had less effect on HLF while the average MW for COS was more important.

### Fluorescence spectroscopy evaluation for the effect of COSs on the structure of HLF

Fluorescence quenching of proteins is an effective method to determine the structural changes of polysaccharides when interacting with proteins. Figure 1D–F show the fluorescence intensity of HLF was affected by the concentrations of all three COSs. Protein fluorescence quenching is a reduction in the fluorescence intensity of the protein. It is caused by various molecular interactions that result in a decrease in the quantum yield of fluorophore fluorescence [35].

The maximum emission wavelength (λ_max_) of fluorescence for HLF in the absence of COS was around 330 nm, as shown in Fig. 1D–F. This result was consistent with previous studies [29]. At the COSs : HLF concentration ratios of 4 : 1, the fluorescence intensities of COS1–HLF, COS2–HLF, and COS3–HLF complexes were 622, 658 and 662, respectively. COS1 had the most evident effect on the fluorescence quenching of HLF. COS1 and COS2 originated from shrimp and crab shells, but COS1 had a smaller average MW. Therefore, the lower the MW of COS was, the greater the interaction effect on HLF. Compared with COS2 and COS3, when the COS concentrations were relatively lower in the mixture (COSs : HLF concentration ratios from 1 : 8 to 1 : 1), COS3 had a higher effect than COS2 on the fluorescence quenching of HLF. For example, when the COSs : HLF concentration ratio was 1 : 2, the maximum absorbance of COS2–HLF and COS3–HLF complexes were at 825 nm and 781 nm, respectively. However, when the COSs : HLF concentration ratio reached 2 : 1, the maximum absorbance of COS2–HLF and COS3–HLF complexes was 729 nm and 738 nm, respectively. No significant differences were observed for fluorescence quenching on HLF. Although the MWs of COS2 and COS3 were almost the same, COS3 was derived from *Aspergillus ochraceus*, which had a different crystalline structure from COSs derived from shrimp and crab shells [37]. Therefore, the conformational difference of COS also influenced the interaction effect on HLF.

### Analysis of binding parameters between COS and HLF

#### Mechanism of the quenching of COSs‐HLF complexes

**Fig. 2:**
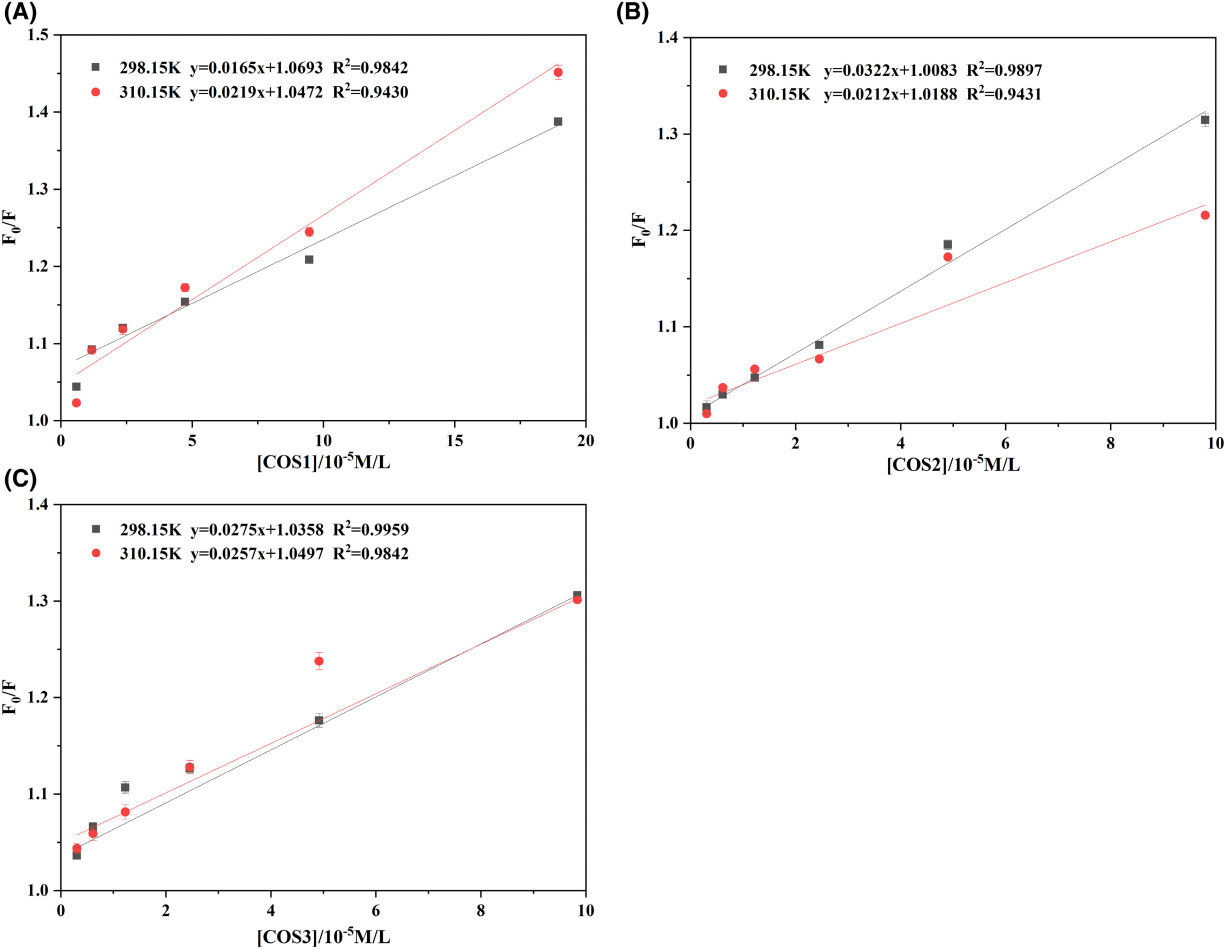
Stern–Volmer analysis for the fluorescence quenching of HLF by COS1 (A), COS2 (B), COS3 (C) at different temperatures.

The two major quenching of fluorescence emission mechanisms are classified as static quenching and dynamic quenching. Typically, static quenching originates from the formation of nonfluorescent ground state complexes, whereas dynamic quenching is from a collision between the fluorophore and the quencher [36]. To confirm the mechanisms of the quenching of the COSs–HLF complexes, the temperature dependence of the Stern–Volmer quenching constant was investigated (Fig. 2). If the values of $K_{\mathrm{sv}}$ (calculated by slopes in Fig. 2) increase with the increasing of temperature, it is associated with a dynamic quenching; otherwise, it is a static quenching. $K_{\mathrm{sv}}$ and $K_{q}$ could be calculated using Eq. (1). Table 2 shows the $K_{q}$ of all COS–HLF complexes exceeded 2 × 10^10^ L·mol^−1^·s^−1^. It meant the static quenching was dominant in COSs–HLF complexes [38]. For COS2–HLF and COS3–HLF complexes, a higher temperature resulted in a lower value of $K_{\mathrm{sv}}$. This result was strong evidence for static quenching. However, the values of $K_{\mathrm{sv}}$ for COS1–HLF complex increased with the increasing of temperature, indicating the simultaneous presence of dynamic quenching for COS1–HLF. This finding suggested that the COS MWs influenced the quenching mechanism of COSs–HLF complexes.

**Table 2:** Bimolecular quenching constant (*K*_q_), binding constant (*K*_a_), and binding point (*n*) of the three COSs with HLF at different temperatures.

Complexes	*T*/(K)	*k*_q_ ± SD/(×10^11^·m ^−1^)	*R* ^2^	*K*_a_ ± SD/(L·mol^−1^)	*n* ± SD	*R* ^2^
COS1–HLF	298.15	1.654 ± 0.001	0.9842	37.186 ± 0.201	0.538 ± 0.050	0.9669
	310.15	2.192 ± 0.002	0.9430	78.679 ± 0.251	0.610 ± 0.061	0.9617
COS2–HLF	298.15	3.215 ± 0.002	0.9897	1413.416 ± 0.214	0.909 ± 0.049	0.9883
	310.15	2.118 ± 0.002	0.9431	124.940 ± 0.347	0.687 ± 0.081	0.9468
COS3–HLF	298.15	2.748 ± 0.002	0.9959	74.210 ± 0.194	0.595 ± 0.043	0.9917
	310.15	2.571 ± 0.003	0.9842	54.465 ± 0.180	0.563 ± 0.045	0.9753

#### Binding properties between COSs and HLF

When COSs as small molecules bind independently to the set of equivalent sites on HLF, the binding constant ($K_{a}$) and the number of binding sites (*n*) can be calculated by Eq. (2). Table 2 shows COS1–HLF complex had the largest $K_{a}$ value at the temperature of 310.15 K. The result indicated that a relatively high temperature improved the stability of COS1–HLF complex. The reaction between COS1 and HLF was a heat‐absorbing process. For COS2–HLF and COS3–HLF complexes, the maximum $K_{a}$ values were at 298.15 K. The reactions were exothermic processes. These results demonstrated that the temperature affected the binding stability of COS–HLF complexes. Furthermore, the MWs (Table 1) of COS2 and COS3 (chitopentaose) were higher than COS1 (a mixture of chitobiose and chitotriose). The different reaction processes between COSs and HLF demonstrated that the MWs of COSs also influenced the binding between COS and HLF.

In our study, COS–HLF complexes were always presented in solution, and no precipitation was observed. We determined the thermodynamic parameters of the complexes and studied the noncovalent interactions of the complexes to elucidate the binding properties of COS to HLF. Protein and polysaccharide complexes in aqueous media are generally driven by hydrogen bonding, hydrophobic interaction, Van der Waals forces, and intermolecular electrostatic forces to form complexes [30, 39, 40]. Table 3 shows the ΔH > 0 and ΔS > 0 at temperatures of 298.15 K and 310.15 K meant the formation of COS1–HLF complex mainly relied on hydrophobic binding. In the COS structure, the –CH and –CH_3_ groups were hydrophobic [30]. Therefore, HLF might bind to these groups in COS1 hydrophobically. For COS2–HLF and COS3–HLF complexes, the results of ΔH < 0 and ΔS < 0 indicated the formation of COS2–HLF and COS3–HLF complexes were mainly Van der Waals forces or hydrogen binding. Furthermore, ΔG < 0 in the three COS–HLF complexes demonstrated that the binding was spontaneous [41]. In brief, the type of force between COSs and HLF was influenced by the MWs but not the crystalline structure of COSs.

**Table 3:** Enthalpy change (Δ*H*), free energy change (Δ*G*), and entropy change (Δ*S*) of the three COSs with HLF at different temperatures.

Complexes	*T*/(K)	Δ*H*/(kJ·mol^−1^)	Δ*G*/(kJ·mol^−1^)	Δ*S*/(J·mol^−1^·K^−1^)	Binding forces
COS1–HLF	298.15	48.015	−8.963	191.105	Hydrophobic forces
	310.15		−11.257	191.108	
COS2–HLF	298.15	−155.422	−17.981	−460.979	Hydrogen bond and van der Waals force
	310.15		−12.449	−460.980	
COS3–HLF	298.15	−19.819	−10.676	−30.666	
	310.15		−10.308	−30.666	

### Molecular docking to determine the binding sites of COS on HLF

**Fig. 3:**
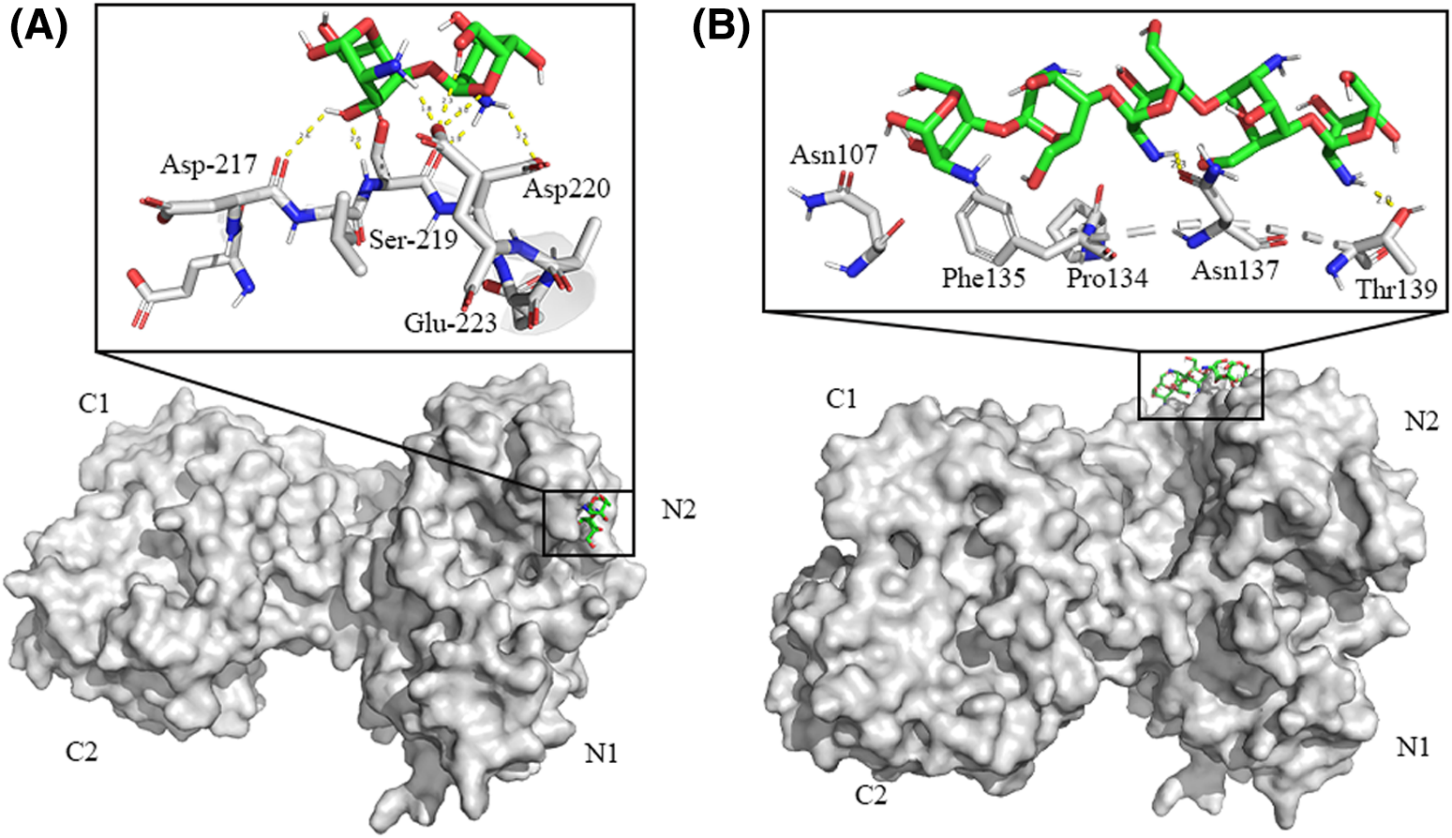
Surface diagrams of HLF with chitobiose (A) and chitopentaose (B). The spheres' model is HLF, and the sticks' model is COSs. The inset is the enlarged drawing of the predicted high‐affinity pocket.

The possible binding sites of COS on HLF were modeled using molecular docking. The conformation with the least free energy, which should be close to the experimental free energy, was chosen based on the results of the study (Fig. 3). The binding force of chitobiose to HLF was −2.52 KJ·mol^−1^. The amino acids bound to chitobiose were Asp217, Ser219, Asp220, and Glu223, surrounding with hydrophobic amino acids such as Val214, Phe215, Leu218, and Ala222 (Fig. 3A). Chitobiose was mainly bound to the N2 domain of HLF. The binding energy of chitopentaose to HLF was 3.45 KJ·mol^−1^. The amino acids bound to chitopentaose were Asn107, Pro134, Phe135, Asn137, and Thr139, as shown in Fig. 3B. Chitopentaose was also bound to the N2 domain of HLF. Based on the data of binding energies, chitobiose–HLF complex was more stable than chitopentaose–HLF. This result was consistent with the UV and fluorescence determination results.

## Discussion

Chitosan oligosaccharide is an oligomeric mixture of glucosamine, which has no UV absorption from 190 nm to 500 nm. Lactoferrin has an UV absorption peak at 280 nm mainly due to the phenyl groups of Trp, Tyr, and Phe [32]. With the addition of COSs, the significant change in the UV absorption of HLF at 280 nm was due to the exposure of more Trp, Tyr, and Phe residues in the structure of HLF to the environment [32]. The result meant the COSs could affect the tertiary structure of HLF. The blue shift in the absorption peak caused by COS1 indicated that COS1 induced peptide chain stretching of HLF molecules, as shown in Fig. 1A. The exposure of Tyr, Trp, and Phe residues within the HLF molecules caused a conformational change of the protein molecule.

The intrinsic fluorescence of HLF was mainly contributed by the Trp residue alone, since Phe and Tyr have a very low quantum yield [42]. If COS binding occurs close to the location of Trp residues in HLF, fluorescence quenching can be observed [30]. Therefore, the emission wavelength of fluorescence from Trp residues can be used to study changes in the local microenvironment of Trp, revealing the effect of COSs on HLF conformational changes [31]. In our experiments, as shown in Fig. 1D–F, fluorescence intensity decreased because the binding of the three COSs resulted in the fluorescence quenching of HLF. Then, the extensive exposure of hydrophobic groups changed the tertiary structure of HLF.

COS2 and COS3 had a static quenching on HLF (Table 2). COS1 interacted with HLF such that static and dynamic quenching occurred simultaneously (Table 2). Typically, static quenching is due to the formation of a nonfluorescent state complex, but dynamic quenching comes from the collision between the fluorophore and the quencher [36]. This collision caused the Trp residues (the main source of the intrinsic fluorescence for HLF) to return to the ground state from the excited singlet state with a radiation‐free leap. After that, the fluorescence intensity of HLF was decreased. Moreover, the higher the concentrations of COSs were, the lower the fluorescence intensity of HLF, indicating more collisions between COSs and Trp residues in HLF.

The molecular docking results verified that smaller MWs of COSs could form more stable binding of COSs to HLF. This analysis was consistent with our experimental results. Therefore, COS MWs and the concentrations had influence on the interaction between COS and HLF.

It was reported that source and treatment of chitosan had effect on its crystallinity, and further affected its characteristics [43]. Chitosan originated from shrimp and crab shells or *A. ochraceus* had different crystal structures [37], so COSs hydrolyzed by them to the same MWs (COS2 and COS3) had the same interaction mechanism with HLF. Only at relatively low COS concentrations in the COS–HLF mixture, the fluorescence quenching effect of COS derived from *A. ochraceus* on HLF was higher than that derived from shrimp and crab shells. These results demonstrated that different crystalline structures of COSs did not have significant influence on the interaction between COSs and HLF. However, under the same MWs, COS derived from *A. ochraceus* had better effect on HLF than COS from shrimp and crab shell. Our study suggested that COSs, especially low MWs, could be a good candidate to remove lactoferrin from tear proteins in contact lens care solutions.

## Conclusion

In summary, the interaction mechanism between HLF and COSs from different crystalline structures and MWs was analyzed. The MWs and concentrations of COSs were key factors for the formation of COS–HLF complexes. The smaller the MWs of COS were, the more stable the binding of the complexes. The crystalline structures of COSs had less impact on the formation of COS–HLF complexes. However, under the same MWs, COS from *A. ochraceus* had a greater effect on HLF compared with COS from shrimp and crab shell. Therefore, adding low MWs of COS from *A. ochraceus* to the contact lens care solutions could improve the HLF removal effect.

## Conflict of interest

The authors declare no conflict of interest.

## Author contributions

QL and SR conceived and designed the study. JL, SG, and BC performed the experiments. JL, SG, BC, QL, and SR analyzed the data. JL and QL wrote the original draft. SG and SR revised the paper. QL and SR supervised and acquired funding. All authors reviewed the results and approved the final version of the manuscript.

## Data Availability

The datasets used and/or analyzed during this study are available from the corresponding author upon reasonable request.
